# Dafadine Does Not Promote Dauer Development in *Pristionchus pacificus*

**DOI:** 10.17912/micropub.biology.001470

**Published:** 2025-01-22

**Authors:** Heather R. Carstensen, Ray L. Hong

**Affiliations:** 1 Biology Department, California State University, Northridge, Northridge, California, United States

## Abstract

In response to unfavorable conditions, nematodes develop into the stress-resistant dauer larvae. Under favorable conditions, many nematodes are known to synthesize dafachronic acids (DAs) that bind to the conserved nuclear hormone receptor DAF-12 to suppress dauer development. However, the enzymes involved in the production of DAs have not been thoroughly investigated in *Pristionchus pacificus* . Here we show that the cytochrome P450 inhibitor Dafadine-A, which suppresses DAF-9 in DA biosynthesis in *C. elegans* and other nematode species, does not cause constitutive dauer formation or gonad migration defects in *P. pacificus* wild type. Instead, Dafadine-A may slightly reduce *P. pacificus* growth rate.

**Figure 1. Dauer formation in C. elegans and P. pacificus following Dafadine treatment:**
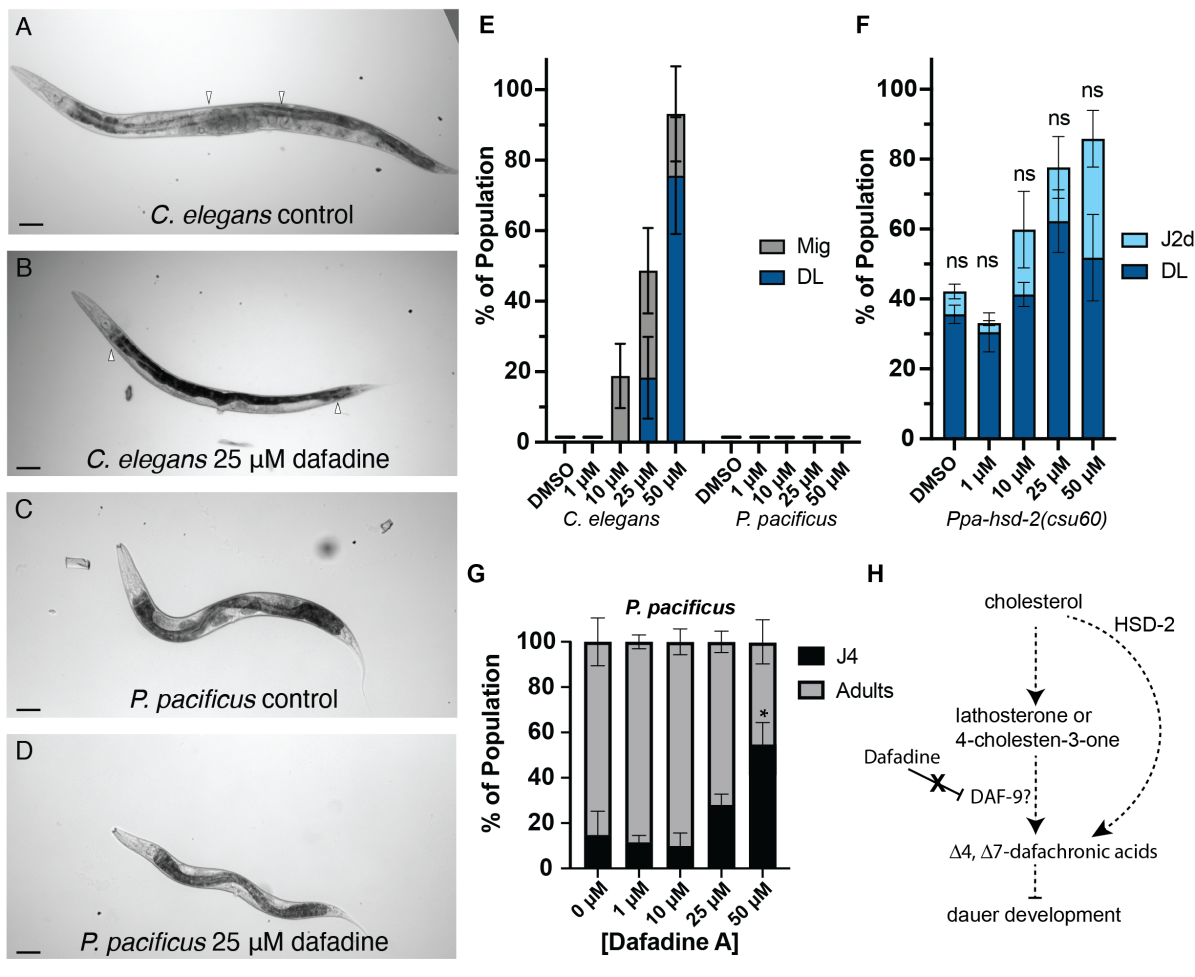
**(A)** *C. elegans* adult cultured on DMSO control plate. **(B)** Adult *C. elegans* cultured on 25 µM Dafadine exhibits characteristic protruding vulva (Pvl) and defect in the migration of distal tip cells in the gonad (Mig) phenotypes, in addition to the Daf-c dauer larvae (DL) phenotype. White arrows indicate the distal tip cell location. Scale bar represents 50 µm. **(C-D)** Compared to *P. pacificus* adults cultured on DMSO, adults on 25 µM Dafadine do not show Mig or Pvl phenotypes. **(E)** Synchronized eggs on DMSO control or Dafadine plates were scored for Daf-c DL and Mig phenotypes on Day 4. **(F)** Dafadine also did not enhance the J2 pre-dauer (J2d) or Daf-c dauer larvae (DL) phenotype of *Ppa-hsd-2 ( csu60 )* on Day 5. *P* >0.05 Dunnett's multiple comparisons test to 0 µM Dafadine. **(G)** The developmental rate of wild-type *P. pacificus* shown in (E) was noticeably delayed at the highest concentration of Dafadine. * *P* <0.05 Dunnett's multiple comparisons test to 0 µM Dafadine. Error bars indicate the standard error of the mean. A minimum of 3 assays were performed per condition. **(H)** A working model of dafachronic acid production from dietary cholesterol in *P. pacificus* .

## Description

Organisms can undergo developmental arrest to cope with unpredictable changes in environmental condition. Almost all nematodes contain the capacity to develop into the developmentally arrested dauer larval stage or the equivalent infective juveniles in parasitic species. In *Caenorhabditis elegans* , the dauer larva is a non-feeding diapause stage induced by starvation, high temperature, and high population density. Specifically in *C. elegans* , dauer formation is induced by low levels of signaling from the TGF-ß and insulin/IGF(IIS) pathways, which were identified by forward genetic screens for dauer formation constitutive mutants (Daf-c) (Albert and Riddle 1988; Malone and Thomas 1994) . Thus, loss-of-function *daf-7* (TGF-ß ligand) and *daf-2* (insulin receptor) mutants form constitutive dauers even under well-fed, low-population density conditions (Ren et al. 1996; Schackwitz et al. 1996) . During non-stressful reproductive development, the TGF-ß and insulin/IGF(IIS) pathways promote the biosynthesis of steroid hormones, primarily Δ7-dafachronic and Δ4-dafachronic acids (DA), which bind to the nuclear hormone receptor DAF-12 to suppress dauer formation (Antebi et al. 1998; Motola et al. 2006) . However, the degree of conservation in the genes involved in dauer formation in other nematodes has not yet been studied extensively.

Surprisingly in the entomophilic nematode *Pristionchus pacificus ,* defects in several well-studied *C. elegans* Daf-c homologs do not show dauer formation defects. For genes with 1-1 orthologs, defects in the RFX master regulator for ciliogenesis, *daf-19* , or in the guanynyl cyclase, *daf-11* , do not produce the Daf-c phenotype in *P. pacificus* (Moreno et al. 2018; Lenuzzi et al. 2021) . For Daf-c genes with 1-many homologs, single, double, and quadruple mutants in the seven *P. pacificus* *daf-7* paralogs do not exhibit Daf-c phenotypes (Lo et al. 2022) . Consistent with the lack of TGF-ß signaling in dauer formation, loss of the four *P. pacificus* *daf-3* paralogs or the three *daf-4* paralogs alone or in combination also do not result in dauer regulation defects (Lo et al. 2022) . In contrast, a null mutation in the sole hydroxysteroid dehydrogenase homolog, *Ppa-hsd-2 ( csu60 ),* results in a severe Daf-c phenotype with about half of the population in the J2d or dauer stage in well-fed OP50 cultures (Carstensen et al. 2021) . In comparison, single mutants of the three *hsd* paralogs in *C. elegans* do not result in Daf-c phenotypes on well-fed plates (Patel et al. 2008; Dumas et al. 2010; Farris et al. 2019) . Because HSDs are involved in the biosynthesis of dafachronic acids from dietary cholesterol, and yet the null allele *Ppa-hsd-2 ( csu60 )* does not completely abolish all non-dauer development, other steroidogenic enzymes must also be involved in the production of steroid hormones as ligands for DAF-12 in *P. pacificus* (Mahanti et al. 2014) .

One likely parallel DA biosynthesis pathway involves homologs of DAF-9 , a cytochrome P450 enzyme, that functions downstream of short chain dehydrogenases DHS-16 and Rieske-like oxygenases DAF-36 (Albert and Riddle 1988; Gerisch et al. 2001; Rottiers et al. 2006; Wollam et al. 2012) . Since there are three *daf-9* -like genes in the *P. pacificus* genome (PPA15273, PPA25583, PPA12512), we wondered if we could determine the presence of DAF-9-like P450 enzymes involved in promoting dauer entry by inhibiting their activity using small-molecules. Dafadine has been identified as a compound capable of inhibiting DAF-9 activity in various nematode species including *C. elegans , Haemonchus contortus ,* and *Auanema freiburgensis* (Luciani et al. 2011; Adams et al. 2019; Ma et al. 2019) . To target potential DAF-9-like enzymes involved in *P. pacificus* dauer formation, we cultured wildtype *C. elegans* and *P. pacificus* on Dafadine-A and looked for Daf-c phenotype. We found that *C. elegans* on Dafadine exhibited gonad migration (Mig) and protruding vulva defects (Pvl) in addition to constitutive dauer formation (including partial L2d) in a concentration-dependent manner as expected (Jia et al. 2002; Luciani et al. 2011) . However, these phenotypes, including partial dauers characteristic of *C. elegans* *daf-9* mutants (Gerisch et al. 2001; Zhang and Sternberg 2022) , were not observed in wild-type *P. pacificus* ( **Figure 1A -E** ). Moreover, Dafadine did not enhance the partial J2d pre-dauer or complete Daf-c phenotype of *Ppa-hsd-2 ( csu60 )* mutants ( **Figure 1F )** . The highest Dafadine concentration (50 µM) could only slightly retard the developmental rate of wildtype *P. pacificus* at ( **Figure 1G ),** although this effect could be also due to the 5x greater volume of DMSO present in the 50 µM Dafadine plates rather than the Dafadine itself. Thus, in *P. pacificus,* Dafadine appears to have very limited inhibitory activity against certain cytochrome P450 enzymes involved in development and growth but not against DAF-9-like homologs, if there is any, involved in dauer formation **( Figure 1H )** .

Our findings add to the suspicion that while the developmental logic may still be preserved in dauer development in *P. pacificus* , canonical pathways known to mediate the sensory and dietary cues processed by the endocrine pathway in *C. elegans* have undergone significant developmental system drift (True and Haag 2001; Sommer 2020) . Future studies could focus on determining the precise ensemble of genes that collaborate with *Ppa-* HSD-2 in DA biosynthesis, as well as upstream factors that converge onto the DAF-12 molecular switch.

## Methods

**Strains and maintenance**

Nematodes were cultured on OP50-seeded NGM Lite (34.22 mM NaCl, 22.04 mM KH _2_ PO _4_ , 2.87 mM K _2_ HPO _4_ , 4 g/L Bacto-tryptone, 20 g/L Bacto-agar, 12.93 µM cholesterol) plates and assayed at 20°C (Brenner 1974) .

**Dafadine assay**

Dafadine-A (>98%; CAS: 1065506-69-5 from Arctom Scientific) stock solution (10 mM) was prepared with DMSO. Dafadine-A stock was added to autoclaved NGM Lite media after autoclaving to reach 1 µM, 10 µM, 25 µM, or 50 µM concentrations, and poured into 6 cm plates. DMSO vehicle control was 0.1% (v/v), an equivalent volume to the 10 uM dafadine-A plates. Plates were stored at 4 **°** C, then seeded with 150 µl OP50 two days before commencement of the assay. Only freshly poured Dafadine-A plates less than 3 weeks old contributed to our findings, given that there was a noticeable significant reduction in efficacy in the *C. elegans* controls beyond 3-week old Dafadine plates. To set up assay, five *C. elegans* N2 adult hermaphrodites, or 10-15 *P. pacificus* PS312 adult hermaphrodites were were allowed to lay eggs on 2-day old OP50 lawns for 4-5 hours at 22 **°** C. The synchronized eggs were incubated at 20 **°** C for 4-5 days when the progeny populations were scored for developmental stage, Daf-c dauer phenotype, and Mig phenotype.

**Microscopy**

Representative DIC images were captured with a Leica DM6000 with a 40X oil objective. Animals were mounted on a 3% agar pad on microscope slides, and anesthetized with 77 mM sodium azide in M9 buffer.

## Reagents

**Table:** 

Strain name	Genotype
N2	*C. elegans* wild type
PS312	*P. pacificus* wild type
RLH240	*P. pacificus* *Ppa-hsd-2 ( csu60 ) *
